# Supplementary material for: Are carbon emissions trading and green financial instruments synergistic? -Comprehensive quantitative research based on content analysis
Source: PLoS One. 2024 Mar 7;19(3):e0298601. doi: 10.1371/journal.pone.0298601 (PMC10919601; doi:10.1371/journal.pone.0298601)
Supplement: S1 Data — (ZIP) [file pone.0298601.s001.zip › Data/地级市Z_F工作报告.zip!/╣π╬≈/║╪╓▌╩╨/└┤╫╘╣1⁄2╓┌║┼í╛┬φ┐╦╩2╛▌═°í┐_2014.docx]

各位代表：

现在，我代表市人民政府向大会作政府工作报告，请予审议，并请市政协委员和列席会议的同志提出意见。

一、2013年工作回顾

过去一年，面对宏观经济的复杂形势和下行压力，在自治区党委、政府和市委的正确领导下，我们坚持稳中求进的工作总基调，团结带领全市人民，沉着应对风险挑战，全面推进科学发展，重点工作亮点频现，各项事业取得了新的进步。

——经济总量有新增长。初步统计，全市实现地区生产总值423.9亿元，增长8.7%。财政收入35.76亿元，增长11.6%。公共财政预算支出突破百亿元大关，达107.3亿元，增长9.8%。各县（区、管理区）全部迈上地区生产总值50亿元台阶。

——产业结构有新优化。二、三产业比重达78.2%。其中，规模以上工业增加值超百亿元，达115亿元，增长16.5%；接待游客超千万人次，达1030万人次，增长26.8%；旅游总收入超百亿元，达101.6亿元，增长39.3%。旅游总收入占地区生产总值比重超过20%。

——重点企业有新贡献。兆鑫五金制品公司产值达42.77亿元，被评为自治区强优企业。华润（贺州）电厂产值达48亿元。信都工业区产值已超百亿元，达110亿元。全市规模以上工业企业利润达22.1亿元。

——居民收入有新提升。全面实施财政收入、城镇居民收入、农民收入“三个倍增计划”，城镇居民可支配收入21682元，增长9.2%；农民人均纯收入6557元，增长12.6%。

——多项工作有新佳绩。广贺高速公路全线通车，出省通道建设实现新突破。贺州被国家发改委确定为全国31个资源成长型城市之一；荣获“国家森林城市”称号；成功举办富川瑶族自治县30周年县庆；华润循环经济产业示范区成为“国家循环经济教育示范基地”。

一年来，我们主要做了以下工作：

（一）抓投资扩规模，特色优势产业体系不断壮大。重大项目稳步推进。全社会固定资产投资483.2亿元，增长18%。争取到中央、自治区补助资金项目417项，获国家资金12.17亿元。金利稀土新材料、扬翔饲料、平桂碳酸钙新材料产业园标准厂房等一批重大项目开工建设。华润雪花啤酒、漓源食品、恒希陶瓷、高峰桂山等一批重大项目竣工。投资500万元以上项目竣工1187项，自治区和市级层面重大项目分别完成投资31.8亿元和140亿元。支柱产业规模扩张。新增规模以上工业企业12家，规模以上工业企业达170家，产值超亿元企业达62家。电力、林产、矿业、电子、新材料和碳酸钙、食品药品等支柱产业完成工业总产值319亿元，占全部工业总产值的74%。农业产业化提速。粮食总产量达73.55万吨，实现“十连增”。在全区率先启动实施厅市共建农业标准化示范市工作，推广标准化生产技术面积71.5万亩。八步区、平桂管理区成功创建国家级出口食品农产品质量安全示范区。富川脐橙、英家大头菜、贺街淮山、芳林马蹄获得农业部农产品地理标志登记保护，昭平茶获得质检总局中国地理标志产品保护。完成造林面积30.9万亩，林下经济产值达17.99亿元。新增规模农业企业5家、专业合作社60家。全市农林牧渔业总产值达146.41亿元，增长4.5%。旅游带动效应明显。出台实施加快旅游业跨越发展的扶持政策，举办2013′贺州特色美食评选活动，姑婆山国家森林公园创建国家生态旅游示范区工作通过验收，南乡森林温泉度假村项目顺利推进。在旅游业带动下，零售、物流等服务业蓬勃发展，社会消费品零售总额达119亿元，增长11.9%。

（二）护生态建新城，城市综合服务功能不断完善。生态保护成效显现。“绿满八桂”工程持续推进。加强对姑婆山等五个自然保护区生态环境保护，昭平七冲自然保护区晋升为国家级保护区，龟石水库湿地公园项目通过国家评审。在全区率先开展“清洁城镇乡村，建设美丽贺州”活动，探索出城乡环境卫生管理的好措施、好机制，城乡环境卫生面貌有所改观。果断应对贺江水污染事件，仅用14天时间就终止了Ⅱ级突发环境事件应急响应，并铲除近千家作坊式的“五小”窝点，马尾河、西湾河等水质明显改善。试行水源地生态补偿机制和市县乡三级“河长制”管理制度。建成生态文明新农村示范点35个。昭平县、钟山县荣获“全国生态文明先进县”称号，富川瑶族自治县荣获“自治区循环经济示范县”称号。交通路网构建提速。景观大道、西南环路建成投入使用，贺州至姑婆山公路改扩建工程竣工。贵广高铁贺州段、平桂至富川一级公路（平桂至望高段）、贺州港马江作业区建设稳步推进，北环路、国道323贺州至钟山段改造工程、大汤经南乡至广东福堂二级公路等项目前期工作加快推进，贺州支线机场项目被国家民航部门列为 “十二五”新建项目。城镇基础设施改善。启动编制“生态新城”核心区概念性规划。望高至担杆岭水厂第二条原水输水管道、路花水库第二水源工程等项目前期工作扎实推进，中心城区及各县城的城镇污水生活垃圾处理设施建设工程竣工并投入使用，城区供水保障能力和污水收集处理率逐步提高。依法集中拆除城东新区连片“两违”建筑，绿洲家园、贺州大酒店、城东新区路网工程等重点项目加快推进，31个重点城建项目完成投资11.02亿元。中心城区建成区面积扩至36平方公里，各县城、建制镇建设水平进一步提升，全市城镇化率达41%。

（三）抢机遇扩开放，发展的动力与活力不断增强。开放型经济逆势攀升。完成粤桂产业合作示范区发展规划编制，桂贺一体化发展规划通过自治区发改委评审。成功举办第七届广西贺州农产品展销会、贺州市（南安水头）石材产业推介会。与华润集团、中国冶金地质总局、中国港中旅集团公司、中国广核集团等知名企业的合作项目加快推进。招商引资（含续建）项目到位资金311亿元；实际利用外资9740万美元；进出口总额达1.99亿美元，增长27.9%。各项改革稳步推进。低丘缓坡开发试点、小微企业标准厂房建设用地试点等项目获自治区批准，全市累计土地流转面积85万亩。“营改增”试点工作稳步推进。贺达纸业改制工作基本完成。深入推进“扩权强县”，下放管理事项落实到位。食品安全监管职能调整基本完成，监管工作得到强化。县级公立医院改革工作进展顺利。第三批事业单位实施绩效管理工作有序推进。出台旺高工业区、电子科技生态产业园、华润循环经济产业示范区等重点园区财政收入分配办法。整合国有投融资平台，组建广西贺州正赢发展集团。启动实施万家成长型小企业信贷扶持工程，支持农发行贺州支行、建设银行贺州支行升格工作。金融机构存贷款余额分别达404.8亿元和242亿元，分别增长16.1%和18.2%，增速分别排全区第3位和第4位。地方政府性债务余额46.05亿元，负债率为10.9%。行政效能有所提高。市人大代表建议、政协委员提案办结率达100%。实行政府常务会集中学法、重大项目联合审批等制度，强化行政复议审理及规范性文件的合法性审查。精简市级审批事项113项，积极开展“政务公开日”活动，严格政府采购招投标机制，“一服务两公开”向基层延伸，实现乡镇（街道）政务服务中心全覆盖。

（四）惠民生强保障，社会建设治理水平不断提升。民生投入持续增加。全市民生领域财政支出84.8亿元，占公共财政预算支出的79%。其中，筹措资金20.8亿元兴办10大项48小项惠民工程，除1个项目因受贵广高铁建设影响暂时无法完成外，其余均已完成。社会保障体系不断完善。城镇新增就业16897人，农村劳动力转移新增就业28118人，城镇登记失业率3.56%。稳步推进“诚信计生”、“幸福计生”工作，荣获全区人口和计划生育工作特别先进奖，并在全区率先完成免费孕前优生健康检查任务。社会保险覆盖面不断扩大，城乡居民养老参保率达95.5%，基础养老金发放率达100%。新农合参合率达98.95%，城乡居民健康电子档案建档率达86.09%。保障性安居工程建房开工5462套，新增发放廉租住房租赁补贴917户，完成农村危房改造1.12万户。新建农村饮水安全工程145项，解决15.7万人饮水不安全问题。市社会福利院、儿童福利院、老年活动中心等项目竣工，贺州被确定为全区农村社区幸福院建设试点市。教育基础持续夯实。农村闲置校舍改建幼儿园和农村小学增设附属幼儿园项目开工267项，已竣工168项，新增公办幼儿园114所，市属小学筹建项目全面启动。贺高迁建、二高扩建、平桂高中迁建等教育项目加快推进。深入推进职业教育攻坚五年计划。贺州学院初步完成教育部本科教学工作水平评估。文化先进城创建步伐加快。市文化中心建设稳步推进，市级“三馆”列入国家“十二五”支持建设项目，85个村级公共服务中心如期竣工。贺街镇成功申报自治区级历史文化名镇。成功组织开展“贺江欢歌”、“我来露一手”等群众性文化活动，客家山歌剧《连心店》荣获第十届中国艺术节“群星奖”。贺州籍运动员钟雪纯在第十二届全运会上勇夺女子自由摔跤55公斤级冠军。创新驱动初显成效。获自治区认定科技研发中心2家、创新型企业2家。长城矿山机械公司被认定为国家高新技术企业，直接参与两项国家标准制定工作。科隆粉体公司参与修订五项国家行业标准。钟山县荣获“全国科技进步考核先进县”称号。社会综合治理持续深入。安全生产形势平稳，及时妥善处置了相关应急事件。全市各级调解组织调解纠纷16380件，调解成功率98%。治安状况呈现发案率下降、破案率上升的态势，“打盗抢、保民安”专项工作成效排全区第2位。

一年来，工会、共青团、妇联、驻梧管理、机构编制、外事侨务、史志、档案、民族宗教、发展研究、机关事务、住房公积金、统计、农机、广电、扶贫、残联、供销合作、二轻联社、工商、质量监督、检验检疫、地震、气象、水文、保险、邮政、烟草、通信、人防、民兵预备役和国防动员等各项工作取得了新成绩。驻贺部队和武警官兵在支持地方发展、维护社会稳定方面做出了积极贡献。

各位代表！过去一年，在严峻复杂的环境下，我们顶住了压力，经受住了考验，虽然增长略低于年初预期，增速有所放缓，但质量效益有所提升，结构有所优化，为我市调结构、促改革、强后劲打下了更坚实的基础，成绩来之不易。这是自治区党委、政府亲切关怀、正确领导的结果，是市委总揽全局、科学决策的结果，是市人大及其常委会依法监督和市政协民主监督的结果，是全市各族人民团结拼搏、开拓进取的结果。在此，我代表市人民政府，向全市广大干部群众，向全体人大代表和政协委员，向各民主党派、工商联、各人民团体和社会各界人士，向驻贺部队和武警官兵，向关心支持贺州发展的老领导、老同志，表示衷心感谢并致以崇高敬意！

同时，我们也清醒地认识到，我市后发展欠发达的基本市情没有改变，经济社会发展中还存在不少困难和问题，主要表现在：经济总量偏小，人均水平较低；产业结构不优，自主创新能力不强；基础设施不完善，城镇化水平不高；改革开放深度广度还不够，一些体制性障碍尚待破解；政府职能需要进一步转变，如去年7月发生的贺江水污染事件，表明我市环境保护累积问题不少，日常监管工作还存在严重的形式主义和官僚主义，教训惨痛而深刻。对此，我们要以对人民高度负责的精神，更加努力做好工作，全力加快解决。

二、2014年工作安排

今年是全面贯彻落实党的十八届三中全会精神的开局之年，也是实现与全国全区同步全面建成小康社会的重要一年。国家全面深化改革扩大开放，大力进行经济结构调整，使经济发展速度与发展质量并重，同时努力保障和改善民生。此外，我们还面临着西部大开发战略深入实施、中国—东盟打造“钻石十年”、国家支持广西打造我国中南西南地区开放发展新的战略支点、珠江—西江经济带发展规划即将上升为国家战略等重大发展机遇。特别是随着我市交通、能源、工业、旅游和生态健康产业集群、城建等一批重要基础设施和重大项目的实施，贺州的生态优势、资源优势、区位优势将加速转化为经济优势，为我市科学发展、加快发展增添新动力。为此，我们必须抢抓机遇，乘势而上，不松劲、不懈怠，坚持发展第一要务不动摇，更加注重改革开放，更加注重发展的质量和效益，更加注重“调结构、转方式、促转型”，更加注重改善民生，更加注重生态和环境保护，实现经济、社会、生态协调发展，努力开创一个更加崭新的局面！

今年政府工作的总体要求是：深入贯彻党的十八大和十八届二中、三中全会以及中央经济工作会议精神，全面落实自治区党委、政府和市委的决策部署，坚持稳中求进工作总基调，把改革创新扩大开放贯穿经济社会发展各领域，以科学发展和改善民生为引领，以建设粤桂产业合作示范区（信都）和生态新城为新的驱动力，着力加强以交通为先导的重大基础设施建设，积极推进新型工业化、新型城镇化和农业现代化，加速转型、保持增速、突出特色、做大总量，当好科学发展的“特长生”和加快发展的“上进生”，努力打造贺州经济社会“升级版”。

今年我市经济社会发展的主要预期目标为：地区生产总值增长10%左右，财政收入增长12.7%，规模以上工业总产值增长16.7%，规模以上工业增加值增长13.5%，全社会固定资产投资增长16%，社会消费品零售总额增长13.5%，居民消费价格指数增幅控制在4 %以内，外贸进出口总额增长13%，城镇居民人均可支配收入增长11%，农民人均纯收入增长13%，城镇登记失业率控制在4.2%以内，城镇化率提高到43%，城镇新增就业人数1.2万人，节能减排指标控制在自治区下达的“十二五”目标年均增幅范围内。

各位代表，实现上述目标，有机遇、有条件、有潜力，但也有挑战、有难度、有压力，我们必须坚定信心，克难攻坚，努力工作，争取更好更快的发展。我们将着重做好以下工作：

（一）以交通能源为重点，推进重大基础设施建设。

基础实则支撑牢。我们要紧抓“高铁时代”的新机遇，突出交通重点，推进能源、水利基础设施建设，进一步夯实发展基础。

坚持交通优先发展。举全市之力加快打造桂粤湘区域性交通枢纽。铁路方面，努力促成今年上半年开通运营南宁至梧州高铁动车，实现贺州经梧州转乘，3个半小时左右到达南宁的目标；协调加快贵广高铁建设，力争年底前建成通车，实现贺州1小时到达广州、50分钟到达桂林的目标，并力争实现开通运营贺州直达南宁高铁动车。同时，抓紧洛湛铁路扩能改造、贺州至梧州城际高铁等项目前期工作，抓好布局合理、便捷通畅的“高铁时代”交通枢纽网络建设。公路方面，加快推进永贺高速公路贺州段、昭平富裕至马江二级公路、国道323线贺州至钟山改造工程、广贺高速贺州东出口至贺街一级公路、大汤经南乡至广东福堂二级公路等项目建设；加快贺州至来宾高速公路、信都至梧州高速公路等项目的前期工作。机场方面，加快落实贺州支线机场选址工作和通用机场前期工作，尽快开展支线机场工程可行性研究，力争在“十二五”期末开工建设。内河水运方面，加快推进贺江扩能工程，疏浚贺江“八步至厦岛段”航道，协调推进桂江航道工程和马江作业区建设，推动航运物流与旅游观光深度融合发展。

提高能源保障能力。启动华润电厂二期、华润富川风电项目前期工作，力争早日开工建设。加强电网建设，重点推进旺高、信都等重点工业园区输变电站和贵广高铁牵引站外部电源配套工程、顺景送变电工程、桂鑫变电站工程、楚越（富川）变电站工程、浦发变电站扩建工程等项目建设。积极推进“市天然气综合利用工程”和“县县通天然气工程”，争取年底实现市区管道供气。抓好电力保障，做好电力供应调度管理；积极探索以发电企业、用电企业为主体的自主协商购电价格机制；试行峰谷电价监管机制，鼓励用电大户错峰用电，确保全年全社会用电量50亿千瓦时以上。

加强水利基础设施建设。开展中小河流治理项目建设，完成9 个治理项目，综合治理河段总长33公里。开展36 座病险水库除险加固工作。抓好中小型灌区节水改造建设。推进农村饮水安全工程建设，着力解决15万人饮水不安全问题。

（二）以现代工业为主导，加快构建现代产业体系。

产业强则贺州强。要加快实施“18812工程”和“十大行动计划”，推动战略性新兴产业发展，促进传统产业改造升级。

加快推进产业园区建设。实施园区建设大会战，坚持新上项目、城市改造迁移项目都要入园建设的原则，鼓励 “园中园”和“飞地工业”等开发建设模式，推进钨钛锡、稀土、碳酸钙产业等特色园区的规划建设。突出抓好华润循环经济产业示范区、电子科技生态产业园、旺高工业区、西湾（平桂）工业区、信都工业区等基础设施建设，通过“筑巢引凤”，力争年内五大工业园区完成基础设施投资20亿元，完成产业项目投资40亿元，园区工业总产值突破300亿元。继续培育具有特色鲜明、竞争力较强的县域产业集群和县域工业集中区，支持八步区建成全市首个工业总产值超200亿元的县域单位。力争全市园区工业总产值占全市工业总产值比重超75%。

壮大提升工业支柱产业。着重巩固提高电力、林产、矿业、电子、新材料等五大优势支柱产业和提升碳酸钙、食品药品产业，确保“5+2”产业实现工业总产值370亿元。碳酸钙产业方面，紧紧抓住自治区将碳酸钙产业纳入千亿元产业的发展机遇，推动贺州市石材产业招商推介会签约项目落地，加快发展高档涂料、人造岗石、纳米碳酸钙材料项目，延伸产业链，力争“十二五”期末碳酸钙产业工业总产值达100亿元。电力产业方面，确保富川龙头、长春风电场（一期）竣工投产，推进富川金子岭、朝东、石家和钟山东岭、昭平北陀等风电场项目建设；林产产业方面，确保高峰桂山刨花板项目达标达产，着力引进高档家具精加工项目；矿业产业方面，着重抓好中冶矿产加工、美仪瓷业异地技改升级、恒希建材二期工程建设；电子产业方面，确保桂东电子三期工程、桂海铝业电子铝箔一期工程、润大科技光盘生产线项目如期新增产值，桂东电子四期、日轻桂银高纯铝、尼吉光电子、百翔硅溶胶蓄电池等项目开工；新材料产业方面，加快金源稀土、金广稀土和金利稀土发展，推进平桂飞碟钨技改项目建设；食品药品产业方面，力争扬翔饲料公司年产20万吨饲料项目竣工投产。同时，抓好新帅数控年产7000台数控设备、钟山正菱机械装备制造等项目，力争装备制造业完成产业投资7亿元以上。要注重推进创新驱动发展，以企业为主体建设技术进步和创新体系。支持华润（贺州）电厂、平桂飞碟、桂东电子等企业创建“两化融合”示范试点。深入实施“质量兴贺”战略，推进广西铝电子质检中心、自治区级重质碳酸钙产品研发检测中心、桂东食品农产品检验检测中心和市食品药品检验所建设。

大力发展现代高效农业。加快构建以农户家庭经营为基础、合作与联合为纽带、社会化服务为支撑的复合型现代农业经营体系。重点扶持农业龙头企业和农民专业合作组织，年内新增规模农业企业5家、农民专业合作社60家。实施“粮安”工程，确保粮食播种面积稳定在205万亩以上、总产量71.5万吨以上。抓好永久性基本农田划定工作，严格执行耕地占补平衡制度，严守全市耕地16.77万公顷耕地保有量和14.41万公顷基本农田保护红线。强化农机基层服务体系建设，农作物综合耕种收机械化水平提高到43.5%。大力发展生态农业，围绕茶叶、烟叶、蔬菜、水果、马蹄、生猪、奶牛、家禽等特色主导农产品生产，推进农业标准化基地建设，联手粤港澳发展一批有机食品、绿色食品生产基地和富硒农产品基地，推进供港蔬菜基地建设。支持钟山县、昭平县创建国家级出口食品农产品质量安全示范区。落实各项强农惠农富农政策，深入推进“万元增收工程”，培育一批万元片区、万元村屯和年收入10万元以上的“示范农户”，拓宽农民增收渠道。

培育发展以旅游、金融为重点的现代服务业。旅游产业方面，紧紧抓住国家建设桂林国际旅游胜地、自治区推动桂贺一体化等重大发展机遇，加快建设华南生态旅游名城和“粤港澳后花园”。以创A级景区为突破口，全面提升旅游景区品质，年内完成黄姚古镇和姑婆山国家森林公园5A级景区提升规划，力争“十二五”期末完成5A级景区创建工作，并积极抓好玉石林、大桂山、秀水状元村、桂江生态园等景区提A和创A工作，推进旅游名县、名镇、名村建设，推动旅游由观光型向深度体验型转变。分批次推进全市56个重点旅游项目，重点推进南乡森林温泉度假村、大桂山旅游开发、贺州·正和城世界风情文化旅游度假区、百姓源文化城、临贺故城保护与旅游开发等16个重大旅游项目建设。力争年内接待游客1180万人次，实现旅游总收入118亿元。金融业方面，深入实施“引金入贺”战略，鼓励发展小额贷款公司、融资性担保公司、村镇银行等金融机构。建立和健全社会信用体系，促进中小企业信用担保体系健康发展。加强保险业参与社会管理工作的探索。规范做好市城投集团12亿元企业债券发行工作，力争融资性担保公司新增担保贷款30亿元以上，小额贷款公司新增贷款3亿元以上。力争年内金融机构存款余额新增50亿元以上，增长12.5%以上；各项贷款余额新增35亿元以上，增长14.5%以上。文化产业方面，加快培育发展文化创意、数字出版、移动多媒体等新兴文化产业，深入挖掘茶文化、莲文化、瑶族文化、客家文化等资源，提高客家山歌剧、瑶族歌舞等创作水平。稳步推进桂台（贺州）客家文化旅游合作示范区建设，打造 “桂台客属联谊会暨客家文化旅游节”等特色文化品牌。

（三）以重大项目为支撑，促进投资平稳较快增长。

项目建设是经济发展的生命线。要进一步抓好项目管理，优化投资结构，确保全年完成全社会固定资产投资560亿元。

统筹推进重大项目建设。完善市四家班子联系重点项目建设制度、“六个一”等行之有效的项目推进模式。实施市级统筹推进重点项目213项，完成年度计划投资157亿元。其中，新开工79项重点项目，力争完成投资72.5亿元；推进74项续建项目建设，力争完成投资68.5亿元；竣工投产项目15项，完成年度投资16.3亿元。

加强项目投资管理工作。继续整合各行业中央及自治区投资的重大项目前期工作、项目建设及后续经营管理，加大百翔公司起动型硅溶胶蓄电池产业化基地等项目前期工作力度，争取年内有实质性进展。高度重视中央预算内投资项目申报工作，提前谋划一批符合中央资金投向的项目，力争全年获中央和自治区项目资金15亿元以上。强化市重点项目建设管理办职能，统筹全市项目联合审批工作，建立项目审批绿色通道，提高项目审批效率。

下大力气抓好要素保障。抓好资金保障，完善“政银企”协调机制，加大信贷投放力度；优化财政支出结构，优先保障重点在建、续建项目资金需求。抓好用地用矿保障，积极开展土地利用总体规划和矿产资源总体规划中期评估及修改工作，探索推行土地利用计划差别化管理。突出工业、产业和城市基础设施建设“三个优先”，统筹保障自治区和市级层面重大项目、重大基础设施、民生工程项目的用地需求。特别要抓好低丘缓坡开发、土地增减挂钩、小微企业标准厂房建设用地和采矿用地改革试点工作。

（四）以东靠战略为导向，掀起开放合作新高潮。

抓住珠江—西江经济带发展规划即将上升为国家战略的有利机遇，深化以东靠战略为重点的开放合作，让开放成为贺州当好科学发展“特长生”和加快发展“上进生”的最强引擎。

规划建设粤桂产业合作示范区（信都）。依托国家级桂东承接产业转移示范区，加强与广佛肇经济合作区、粤桂特别合作试验区的交流合作，加快推动信都工业区扩容提质，高标准规划建设粤桂产业合作示范区。今年要以创新跨省（区）产业发展合作机制为突破口，在信都工业区原有基础上，加快粤桂产业合作示范区合作共建，努力将示范区建成粤桂产业一体化发展的重要平台。重点推进八桂产业区路网工程、信都污水处理厂等基础设施项目，完成投资5亿元以上；开工建设桂航精密铸造等一批产业项目，完成投资9亿元以上，加快形成以信都工业区为核心，辐射灵峰、铺门、仁义、步头等协同发展的工业转型与区域开放相融合的新增长极。

加快拓展更大范围的区域合作。充分利用中国—东盟博览会、美丽广西香港行、桂台经贸合作论坛等平台，拓展与东盟、香港、台湾等地合作交流。同时，积极培育进出口经营主体，加强日用陶瓷、农副产品、工业原材料等外贸企业的跟踪服务。争取新增5家年出口额1000万美元的企业；全市外贸进出口总额突破2亿美元，其中出口总额0.8亿美元，进口总额1.46亿美元。

努力推进更富成效的招商引资。切实加强项目谋划，围绕“5+2”产业和生态健康产业，筛选一批重点产业和产业链条延伸项目向外推介，招引一批关联产业快速聚集，打造我市优势产业集群。进一步完善招商引资工作机制，充分发挥县（区、管理区）、工业园区管委会的积极作用。创新招商引资方式，突出抓好驻点招商、以商招商、节会招商、小分队招商、产业链招商。紧盯行业龙头，抓好与重点企业合作，完善招商项目跟踪服务、督促检查机制，提高招商成功率、合同项目履约率、资金到位率和开竣工率。力争全年招商引资项目到位资金300亿元以上，其中外资项目到位资金9000万美元。

（五）以生态新城为龙头，加快推进新型城镇化建设。

积极稳妥推进以人为核心的新型城镇化，探索实践产城互动发展新路径，强化市中心城区带动作用，加快县城、集镇健康发展，力争全市城镇化率达到43%。

启动实施生态新城规划建设。抓好城市总体规划修编和生态新城总体规划编制工作，按照“特色立城”要求，将生态健康产业集群发展与城市建设有机结合，努力把贺州建设成为生态宜居城市。生态新城规划总面积为70平方公里，核心区规划建设10平方公里。围绕生态旅游观光、生态休闲度假、生态养生保健和生态健康晚年四大业态规划布局生态健康产业集群示范区、贺州园博园、新型特色小镇、都市会展中心、旅游地产、新城中央商务区等产业项目。

加强市中心城区基础设施建设。抓好旧城区排水管网改造工程、市职教中心片区排水工程、市污泥处理厂等项目建设，推进城镇防洪排涝、给排水管网建设。推进望高至担杆岭水厂第二条原水输水管道、担杆岭水厂二期及配套管网、路花水库第二水源工程等项目建设，提高城镇供水能力，确保群众饮用水安全。规划建设火车站公交枢纽，推进北环路、城东新区路网等项目建设，完善城市主骨架路网。完善城东新区水、电、气等配套基础设施建设，增强城市综合服务功能。积极推进太白湖公园、文化中心、文化广场等项目建设。进一步完善市容环境综合整治长效机制，将绿化环卫、城市照明、户外广告等纳入监管范围，加大“两违”查处力度，加强农贸市场环境治理，提升人居环境质量。

突出抓好县城和重点镇建设。大力实施大县城战略，坚持扩容提质和凸显特色并重，强化产业功能、服务功能和聚集功能，有序推进老城改造、新区建设，把各县城建成服务城乡、带动县域的重要节点城市。抓好城区、乡镇、村屯以及县际主要交通沿线、旅游景点周边等层面的风貌塑造，支持贺街镇申报全国历史文化名镇。实施“强镇兴村富民”工程，抓好重点乡镇总体规划修编和控制性详细规划工作，加快推进17个重点镇城乡一体化示范工程，逐步扩权强镇，重心下移，按照“宜工、宜农、宜居、宜游”原则，完善公共基础设施配套建设，培育壮大特色支柱产业，加快向特色小城市转型。

牢固树立以人为核心的新型城镇化理念。积极推进农业转移人口市民化，全面放开建制镇和县城落户限制，有序放开市区落户限制。稳步推进城镇基本公共服务常住人口全覆盖，把进城落户农民纳入城镇住房和社会保障体系，在农村参加的养老保险和医疗保险规范接入城镇社保体系。以社区为依托，建设完善农民工服务和管理平台。

（六）以绿色发展为引领，全面加强生态文明建设。

以刚性约束促进绿色发展、资源节约、环境保护，加快推进生态文明建设，共建环境美好家园。

全面加强生态建设。完善环保考核指标体系和生态补偿机制，深入开展环境安全隐患大清查大整治。推进贺江、桂江沿线6个水质监测自动站建设，加快建立市本级、八步区和平桂管理区固废管理中心，筹建重金属检测实验室，提升环保装备水平和环境检测能力。加大龟石水库等饮用水水源地保护力度，确保饮用水水源水质100%达标。积极创建“广西园林城市”，扎实做好承办广西（贺州）园博会的筹备工作，支持富川争创国家生态文明先行示范区。继续推进“绿满八桂”工程，突出抓好村屯绿化工作，力争森林覆盖率从72.46%提高到72.73%。深入实施“清洁城镇乡村，建设美丽贺州”活动。利用村镇规划集中行动成果，开展村镇环境整治，建设美丽乡村，提升农村环境品质。按自治区的统一部署，及时启动实施“美丽广西”乡村建设第二阶段活动。

强力推进节能减排。大力推广清洁生产技术，减少白色污染、农药和化肥残留污染。继续实施重点节能改造和公共机构节能改造，开展全民节能减排和节能产品惠民行动。强化对全市重点耗能行业和企业的监管，全面执行污染物总量控制和排污许可证制度，加强投资项目节能评估审查管理。落实淘汰落后产能计划，抓好冶炼、水泥等行业的脱硫脱硝工程建设。推进造纸行业提标改造，实施贺达纸业废水深度处理和废气处理改造工程，尽快启动贺达纸业搬迁计划。

大力发展循环经济。深入推广以沼气为纽带的生态循环农业模式，年内新建沼气池5000座，保持农村沼气池入户率居全区前列水平。大力发展新型能源，推进太阳能、风能、小型核电等绿色能源项目建设。全面规范矿产资源开发秩序，实施稀土产业、钨钛产业、林业资源综合利用和平桂资源枯竭型城市产业转型发展等循环经济工程，支持富川争创全国循环经济示范县。

（七）以改革创新为统领，增强经济发展新动力。

要坚决按照中央的统一部署，加快推进重点领域改革，让改革成为发展的最大红利。

加快完善体制机制。加快农村综合改革，探索建立覆盖市县镇村的土地流转综合服务体系，推广富川“白牛模式”、“铁耕模式”等土地流转模式，以集中连片流转为重点，积极稳妥推进土地流转。推动国有企业改革，加快市冶炼总厂改革、市押运公司组建等工作。深化投融资体制改革，发展壮大贺州正赢发展集团。加快事业单位分类改革，推动公办事业单位与主管部门理顺关系和去行政化。深化财税改革创新，推进“营改增”试点工作，稳步实施国家结构性减税政策；想方设法变财政担保制为项目平衡制，最大限度地减轻财政负担；推进市本级预算绩效试点和国库集中支付改革，推进预决算信息公开透明，及时公布2014年度市本级财政预决算“三公”经费；抓好财源培植，强化税收征管，确保财政收入突破40亿元。统筹推进医疗保障、公共卫生、食品药品监管体制等社会领域改革。完善“小财政办大民生”机制，提高教育、医疗卫生、社会保障等支出占地区生产总值比重。

大力发展非公经济。推进实施“抓大壮小扶微工程”和“中小企业成长计划”。鼓励非公企业向专业园区集聚发展；鼓励非公企业参与建设“5+2”现代产业体系；鼓励非公企业参与国企改革和市政设施、医疗事业、教育事业、文化产业等领域建设。落实企业家奖励办法，结合产业人才小高地建设，培养引进一批企业管理和技术人才。完善面向民营企业的投资融资、技术支持、市场开拓、创业服务等公共服务平台，大力培育发展民营经济。全面落实微型企业扶持政策，推行工商登记制度改革，削减资质认定项目。通过产业引导、政策扶持、环境优化等措施，年内发展微型企业605家。

鼓励发展县域经济。完善与主体功能区相匹配的县域经济考核评价体系，推进扩权强县、扩权强镇改革，积极理顺市县乡三级政府间财权、事权关系。依托产业资源优势，加快发展陶瓷、碳酸钙、林产、机械、风电等各具特色的优势产业，加快构建县域特色产业集群。鼓励和支持各县（区、管理区）争创广西科学发展十佳县、进步县。

（八）以改善民生为目的，统筹发展重大社会事业。

坚持把改善民生作为一切工作的出发点和落脚点，强化民生项目投入和推进力度，不断提升人民群众的幸福感和满意度。

完善城乡社会保障体系。落实城乡居民收入倍增计划，使城乡居民收入分别提高到24130元和7440元。加强市县公共就业服务平台网络建设，年内实现农村劳动力转移新增就业2.3万人。加快“全民社保”建设，使城镇五险参保人数累计提高到60.61万人，确保城镇职工医疗保险参保率、城镇居民基本医疗保险参保率、新农合农民参合率稳定在97%以上。加快市老年公寓项目建设，完善社会救助、优抚、救灾、五保供养等社会保障体系建设。

推进社会事业全面发展。深入实施第五轮创新计划，推进科技企业孵化器建设。深入实施中心城区教育项目建设大会战，确保贺州高中在今年秋季学期搬入新校区办学；贺州二高加快扩建，扩大招生规模；市实验中学搬到贺州高中旧址办学；抓紧市属小学的筹办，力争今年秋季学期正式招生办学；八步三中、平桂三中、平桂第一幼儿园投入使用。深入开展“文化先进城”创建活动，继续抓好“扫黄打非”工作，加快市文化中心和市档案馆、图书馆、博物馆、群众艺术馆建设。加快市人民医院城东分院等卫生项目建设，积极争创“全国卫生城市”，稳步推进三县婚育综合服务平台建设。按照国家、自治区统一部署实施好计划生育政策，促进人口长期均衡发展。推进城中新区体育场、游泳馆等体育项目建设，做好承办全区第八届农民运动会筹备工作。抓好全市170个贫困村整村推进扶贫开发工作，深入实施革命老区扶贫攻坚“三年帮扶行动” ，推进省工委革命历史博物馆（钟山）、黄姚古镇广西省工委旧址、古柏山烈士陵园（八步）等项目建设。

维护社会和谐稳定。努力创建“社会和谐稳定模范城”，加强“平安贺州”建设，完善市县两级天网工程建设，集中整治重点地区和突出问题。加强国防动员、国防后备力量建设工作，推进军民融合式发展，积极争创新一轮“全国双拥模范城”。继续推进民族团结进步模范区建设工作。健全乡镇、村、屯三级信访维稳工作网络，扎实推进信访积案化解。全面落实政府安全生产监管责任和企业主体责任，抓好安全隐患排查和整改。强化对公共卫生、食品药品安全、环境安全事件的预防预警、督促检查和应急处置。

实施为民办实事工程。大力筹措资金，继续实施10项为民办实事工程。1.社保惠民。继续做好孤儿生活保障、农村集中养老、城乡医疗救助等工作。加大计划生育特殊困难家庭经济扶助力度。新建一批乡镇中心敬老院、农村社区幸福院和五保村。2.健康惠民。继续实施免费孕前优生健康检查，推进实施地中海贫血防治项目、艾滋病防治项目等。力争全市村级门诊统筹覆盖率达80%以上、村级实行国家基本药物制度达95%以上。3.教育惠民。推进农村义务教育阶段学生营养改善计划，实施中职学生免学费政策。4.强基惠民。加快实施小型农田水利、中小河流治理、病险水库除险加固、农村饮水安全等涉及面广的水利工程建设。5.安居惠民。大力推进保障性安居工程和棚户区改造建设，新建保障性住房3447套，加快解决中低收入家庭住房困难。6.土地整治惠民。继续以申报实施整村推进、整县推进高标准基本农田建设和“小块并大块”的土地整治模式，深入推进农村土地综合整治工作，提高耕地数量和质量。7.农补惠民。全面落实农作物良种补贴、农资综合补贴、农机具购置补贴等各项惠民补贴政策。8.生态惠民。全年完成植树造林28万亩，全民义务植树650万株。9.文化惠民。完成自治区下达的广播电视乡镇无线发射台站和村级公共服务中心新建任务，抓好乡镇、村级农民体育健身工程建设。10.通畅惠民。完成145.6公里的6个县乡道联网沥青（水泥）路工程，116公里的22个通村沥青（水泥）路工程。

三、全面建设人民满意的政府

各位代表，新的一年，我们将以深入开展党的群众路线教育实践活动为契机，巩固和扩大履职尽责“回头看”活动成果，进一步改进工作作风，全面推进法治政府、服务型政府建设，切实做到为人民服务、对人民负责、受人民监督、让人民满意。

（一）加强法治政府建设。扎实开展“六五”普法，深入推进“法治贺州”建设。加强行政执法规划建设，不断提高依法行政水平。建立和完善行政决策风险评估机制，加强重大决策跟踪反馈和责任追究。健全行政复议案件审理机制，纠正违法或不当行政行为。强化政府内部层级监督，自觉接受市人大及其常委会依法监督和市政协民主监督，认真听取各民主党派、工商联、人民团体和社会各界人士的意见。充分发挥监察、审计等专门监督的作用，高度重视人民群众监督和新闻舆论监督。

（二）全面履职主动服务。强化经济运行监测预警，加强对宏观经济形势、就业、物价等情况的研判，及时采取措施加强薄弱环节。强化政府性债务管理，防范债务风险。进一步精简行政审批环节，对仍需保留的行政审批事项实行目录化管理。加强市县乡三级政务服务中心建设，探索帮办、代办、领办等服务机制，年内实现50%村委（社区）建立政务服务站，完成县级电子政务外网横向城域网建设。继续推行“政务公开日”活动，通过便民窗口、热线电话、手机短信等载体，推进政务公开和政府信息公开。加强政府执行力建设，健全行政责任体系，把各项工作落实到单位、部门和具体责任人，推动政府工作高效有序运转。

（三）树立清正廉洁形象。严格遵守执行中央“八项规定”和自治区“两规定一办法”、党政机关厉行节约反对浪费条例、党政机关国内公务接待管理规定等各项政策规定，坚持勤俭办一切事业，坚决遏制“四风”现象。推广政府购买服务，凡属事务性管理服务，原则上都要引入竞争机制。严格控制“三公”经费，压缩一般性支出。严格落实党风廉政建设责任制，坚持反腐倡廉与改革发展力度统一，推进惩治和预防腐败体系建设，严肃查处各类违纪违法案件，切实做到干部清正、政府清廉、政治清明。

各位代表，对党忠诚，对人民负责，全心全意为人民服务是政府的天职。我们一定在自治区党委、政府和市委的坚强领导下，把握改革发展的新机遇，凝聚社会进步的正能量，攻坚克难，创新开拓，努力为全市人民创造更多的福祉！
